# Supplementary material for: Bayesian refinement of protein structures and ensembles against SAXS data using molecular dynamics
Source: PLoS Comput Biol. 2017 Oct 18;13(10):e1005800. doi: 10.1371/journal.pcbi.1005800 (PMC5662244; doi:10.1371/journal.pcbi.1005800)
Supplement: S1 Archive — (BZ2) [file pcbi.1005800.s011.tar.bz2 › gromacs_4.62_bayesian_saxs_refi/share/html/online/grompp.html]

grompp


| grompp Main Table of Contents |

**VERSION 4.6  
Sat 19 Jan 2013**

---

### Description

The gromacs preprocessor
reads a molecular topology file, checks the validity of the
file, expands the topology from a molecular description to an atomic
description. The topology file contains information about
molecule types and the number of molecules, the preprocessor
copies each molecule as needed.
There is no limitation on the number of molecule types.
Bonds and bond-angles can be converted into constraints, separately
for hydrogens and heavy atoms.
Then a coordinate file is read and velocities can be generated
from a Maxwellian distribution if requested.
grompp also reads parameters for the mdrun
(eg. number of MD steps, time step, cut-off), and others such as
NEMD parameters, which are corrected so that the net acceleration
is zero.
Eventually a binary file is produced that can serve as the sole input
file for the MD program.

grompp uses the atom names from the topology file. The atom names
in the coordinate file (option -c) are only read to generate
warnings when they do not match the atom names in the topology.
Note that the atom names are irrelevant for the simulation as
only the atom types are used for generating interaction parameters.

grompp uses a built-in preprocessor to resolve includes, macros,
etc. The preprocessor supports the following keywords:

#ifdef VARIABLE  
#ifndef VARIABLE  
#else  
#endif  
#define VARIABLE  
#undef VARIABLE  
#include "filename"  
#include <filename>

The functioning of these statements in your topology may be modulated by
using the following two flags in your .mdp file:

define = -DVARIABLE1 -DVARIABLE2  
include = -I/home/john/doe  
For further information a C-programming textbook may help you out.
Specifying the -pp flag will get the pre-processed
topology file written out so that you can verify its contents.

When using position restraints a file with restraint coordinates
can be supplied with -r, otherwise restraining will be done
with respect to the conformation from the -c option.
For free energy calculation the the coordinates for the B topology
can be supplied with -rb, otherwise they will be equal to
those of the A topology.

Starting coordinates can be read from trajectory with -t.
The last frame with coordinates and velocities will be read,
unless the -time option is used. Only if this information
is absent will the coordinates in the -c file be used.
Note that these velocities will not be used when gen\_vel = yes
in your .mdp file. An energy file can be supplied with
-e to read Nose-Hoover and/or Parrinello-Rahman coupling
variables.

grompp can be used to restart simulations (preserving
continuity) by supplying just a checkpoint file with -t.
However, for simply changing the number of run steps to extend
a run, using tpbconv is more convenient than grompp.
You then supply the old checkpoint file directly to mdrun
with -cpi. If you wish to change the ensemble or things
like output frequency, then supplying the checkpoint file to
grompp with -t along with a new .mdp file
with -f is the recommended procedure.

By default, all bonded interactions which have constant energy due to
virtual site constructions will be removed. If this constant energy is
not zero, this will result in a shift in the total energy. All bonded
interactions can be kept by turning off -rmvsbds. Additionally,
all constraints for distances which will be constant anyway because
of virtual site constructions will be removed. If any constraints remain
which involve virtual sites, a fatal error will result.

To verify your run input file, please take note of all warnings
on the screen, and correct where necessary. Do also look at the contents
of the mdout.mdp file; this contains comment lines, as well as
the input that grompp has read. If in doubt, you can start grompp
with the -debug option which will give you more information
in a file called grompp.log (along with real debug info). You
can see the contents of the run input file with the gmxdump
program. gmxcheck can be used to compare the contents of two
run input files.

The -maxwarn option can be used to override warnings printed
by grompp that otherwise halt output. In some cases, warnings are
harmless, but usually they are not. The user is advised to carefully
interpret the output messages before attempting to bypass them with
this option.

### Files

| option | filename | type | description |
| --- | --- | --- | --- |
| **-f** | grompp.mdp | Input | grompp input file with MD parameters |
| **-po** | mdout.mdp | Output | grompp input file with MD parameters |
| **-c** | conf.gro | Input | Structure file: gro g96 pdb tpr etc. |
| **-r** | conf.gro | Input, Opt. | Structure file: gro g96 pdb tpr etc. |
| **-rb** | conf.gro | Input, Opt. | Structure file: gro g96 pdb tpr etc. |
| **-n** | index.ndx | Input, Opt. | Index file |
| **-p** | topol.top | Input | Topology file |
| **-pp** | processed.top | Output, Opt. | Topology file |
| **-o** | topol.tpr | Output | Run input file: tpr tpb tpa |
| **-t** | traj.trr | Input, Opt. | Full precision trajectory: trr trj cpt |
| **-e** | ener.edr | Input, Opt. | Energy file |
| **-ref** | rotref.trr | In/Out, Opt. | Full precision trajectory: trr trj cpt |

### Other options

| option | type | default | description |
| --- | --- | --- | --- |
| **-[no]h** | bool | no | Print help info and quit || **-[no]version** | bool | no | Print version info and quit || **-nice** | int | 0 | Set the nicelevel || **-[no]v** | bool | no | Be loud and noisy || **-time** | real | -1 | Take frame at or first after this time. || **-[no]rmvsbds** | bool | yes | Remove constant bonded interactions with virtual sites || **-maxwarn** | int | 0 | Number of allowed warnings during input processing. Not for normal use and may generate unstable systems || **-[no]zero** | bool | no | Set parameters for bonded interactions without defaults to zero instead of generating an error || **-[no]renum** | bool | yes | Renumber atomtypes and minimize number of atomtypes |

---

http://www.gromacs.org  
gromacs@gromacs.org
